# Cytosine Base Editor (hA3A-BE3-NG)-Mediated Multiple Gene Editing for Pyramid Breeding in Pigs

**DOI:** 10.3389/fgene.2020.592623

**Published:** 2020-11-16

**Authors:** Yu Wang, Dengfeng Bi, Guosong Qin, Ruigao Song, Jing Yao, Chunwei Cao, Qiantao Zheng, Naipeng Hou, Yanfang Wang, Jianguo Zhao

**Affiliations:** ^1^ State Key Laboratory of Animal Nutrition, Institute of Animal Science, Chinese Academy of Agricultural Sciences, Beijing, China; ^2^ State Key Laboratory of Stem cell and Reproductive Biology, Institute of Zoology, Chinese Academy of Sciences, Beijing, China; ^3^ College of Life Science, University of Science and Technology of China, Hefei, China; ^4^ Savaid Medical School, University of Chinese Academy of Sciences, Beijing, China; ^5^ Sun Yat-sen Memorial Hospital, Sun Yat-Sen University, Guangzhou, China; ^6^ College of Life Sciences, Qingdao Agricultural University, Qingdao, China

**Keywords:** base editing, NGN PAM, hA3A-BE3-NG, multiple gene editing, pyramid breeding

## Abstract

Pig is an important agricultural economic animal, providing large amount of meat products. With the development of functional genomics and bioinformatics, lots of genes and functional single nucleotide polymorphisms (SNPs) related to disease resistance and (or) economic traits in pigs have been identified, which provides the targets for genetic improvement by genome editing. Base editors (BEs), combining Cas9 nickase and cytidine or adenine deaminase, achieve all four possible transition mutations (C-to-T, A-to-G, T-to-C, and G-to-A) efficiently and accurately without double strand breaks (DSBs) under the protospacer adjacent motif (PAM) sequence of NGG. However, the NGG PAM in canonical CRISPR-Cas9 can only cover approximately 8.27% in the whole genome which limits its broad application. In the current study, hA3A-BE3-NG system was constructed with the fusion of SpCas9-NG variant and hA3A-BE3 to create C-to-T conversion at NGN PAM sites efficiently. The editing efficiency and scope of hA3A-BE3-NG were confirmed in HEK293T cells and porcine fetal fibroblast (PFF) cells. Results showed that the efficiency of hA3A-BE3-NG was much higher than that of hA3A-BE3 on NGH (H = A, C, or T) PAM sites (21.27 vs. 2.81% at average). Further, nonsense and missense mutations were introduced efficiently and precisely *via* hA3A-BE3-NG in multiple pig economic trait-related genes (*CD163*, *APN*, *MSTN*, and *MC4R*) in PFF cells by one transfection. The current work indicates the potential applications of hA3A-BE3-NG for pyramid breeding studies in livestock.

## Introduction

As an agricultural animal, pig is an important meat resource with great economic value. The conventional pig breeding is to pyramid desirable traits by cross breeding with cost and long breeding cycle. The genome-editing technology is an effective approach for pig improvement in growth, meat quality, reproductive capacity, and disease resistance (Song et al., 2020). It is the desired goal to exploit efficient and precise genome-editing tools to achieve rapid pyramid breeding through modifying multiple agriculture-related functional genes simultaneously.

Base editors (BEs), combining Cas9 nickase and cytidine or adenine deaminase, perform efficient and accurate base substitutions (C-to-T, A-to-G, T-to-C, and G-to-A) without double strand breaks (DSBs) at target sites, which provides an alternative strategy for precise genome editing (Komor et al., 2016; Gaudelli et al., 2017). Recently, various versions of BEs were exploited to optimize the specificity, sensitivity, and safety of base conversions (Rees and Liu, 2018). One of the many versions, the hA3A-BE3 system, replaces the rat cytidine deaminase (APOBEC1) with human cytidine deaminase (APOBEC3A), which performs C-to-T conversion more efficiently with expanded activity windows at target sites in human cells, plants, rabbits, and pigs than the original BE3 (Wang et al., 2018; Zong et al., 2018; Liu et al., 2019; Xie et al., 2019). However, the targetable scope of hA3A-BE3 is restricted for use with conventional SpCas9, which recognizes target loci through NGG as its protospacer adjacent motif (PAM) sequence.

This limitation can be overcome by using Cas9 variants with targeting preferences other than NGG PAM, which have been validated using conventional BEs. For example, BE variants, such as VQR-BE3, EQR-BE3, VRER-BE3, SaBE3, and SaKKH-BE3, have been developed to target NGAN, NGA, NGCG, NNGRRT, and NNNRRT PAM sites, circumventing the need for NGG PAM sequences in human cells (Kim et al., 2017). CRISPR-Cpf1-based BEs have even been developed that recognize and target T-rich PAM sequences (TTTV; Li et al., 2018; Kleinstiver et al., 2019). Recently, three newly engineered SpCas9 variants, xCas9, SpCas9-NG, and SpG, were reported to expand the targetable scope of NGN PAM sites in cultured cells, plants, and animals (Hu et al., 2018; Nishimasu et al., 2018; Endo et al., 2019; Fujii et al., 2019; Walton et al., 2020). Further, the SpCas9-NG system has been applied only in bacteria, human cells, plants, and rabbits (Huang et al., 2019; Thuronyi et al., 2019; Wang Y, et al., 2019; Zhong et al., 2019; Li et al., 2020; Liu et al., 2020b).

CRISPR/Cas9 mediated *clusters of differentiation 163* (*CD163*)-deletion conferred the ability of effective resistance to porcine reproduction and respiratory syndrome virus (PRRSVs) infection on pigs (Whitworth et al., 2016; Wang H, et al., 2019). *Aminopeptidase N* (*APN*) gene deletion gave the ability of neonatal piglets to resist infection with the highly virulent transmissible gastroenteritis virus (TGEVs; Luo et al., 2019; Whitworth et al., 2019). For meat production, deletion of the porcine *myostatin* (*MSTN*) gene has been shown to improve muscle growth, resulting in a double-muscled phenotype (Qian et al., 2015). Many of these targeted gene deletions could potentially be achieved by generating a premature terminal codon (iStop-codon) through precise C-to-T mutations *via* cytosine base editors (CBEs; Billon et al., 2017; Kuscu et al., 2017). Precision single-base editing provides a strategy to manipulate functional single nucleotide polymorphisms (SNPs) for accurate genetic improvement in pig production. For example, porcine *melanocortin-4 receptor* (*MC4R*) c.893G>A was reported to be associated with fatness, growth, and feed intake traits (Kim et al., 2000). In the current study, in order to increase the efficiency of base editing at expanded target sites in pigs, hA3A-BE3-NG system was constructed and used to produce C-to-T mutation with high efficiency and expanded editable scope in human cells and porcine cells. Economic related genes including *CD163*, *MSTN*, *APN*, and *MC4R*, were simultaneously targeted *via* hA3A-BE3-NG. To our knowledge, this is the first study to precisely edit multiple genes responsible for economic traits in the porcine genome using BEs, and suggest the incredible potential of using BEs to accelerate molecular pyramid breeding in livestock.

## Materials and Methods

### Plasmid Construction

The hA3A-BE3-NG vector was constructed in this study through in-fusion cloning to transfer the DNA fragment containing VRVRFRR variants of SpCas9-NG from Target-AID-NG (119861#; Addgene, Watertown, MA, United States) to hA3A-BE3 (113410#; Addgene, Watertown, MA, United States). For construction of sgRNAs, oligos were synthesized, annealed, and cloned into the *Bsa*I site of the sgRNA-expressing vector, pGL3-U6-sgRNA-PGK-puromycin (51133#; Addgene, Watertown, MA, United States). The fragment pCAG-tdTomato was cloned into the *Bsp*QI linearized pGL3-U6-sgRNA-PGK-puromycin to construct the sgRNA-tdTomato-expressing vector, pGL3-U6-sgRNA-tdTomato. The sgRNAs used in this study are summarized in Supplementary Table S1. The primers used in the construction of hA3A-BE3-NG are listed in Supplementary Table S2.

### Cell Culture and Transfection

HEK293T cells were cultured in Dulbecco’s Modified Eagle’s medium (DMEM; Gibco, Grand Island, NY, United States), supplemented with 10% fetal bovine serum (FBS; v/v; HyClone, Logan, UT, United States) and 1% Penicillin Streptomycin (v/v; Gibco, Grand Island, NY, United States). HEK293T cells were seeded 1 day prior to transfection in 24-well plates (Corning, Corning, NY, United States), at a density of 1 × 10^5^ cells per well. Cells were transfected with 1 μg base editor plasmid (hA3A-BE3, Target-AID-NG or hA3A-BE3-NG), 500 ng pGL3-U6-sgRNA-PGK-puromycin, and 50 ng pCMV-GFP (11153#; Addgene, Watertown, MA, United States) per well, using Lipofectamine LTX (Life Technologies, Gaithersburg, MD, United States) according to the manufacturer’s recommended protocol. HEK293T cells were cultured at 37°C with 5% of CO_2_.

Porcine fetal fibroblast (PFF) cells were isolated from 35-day-old fetuses of Bama pigs. A day before transfection, PFF cells were thawed and cultured in the Minimum Essential Medium (MEM Alpha; Gibco, Grand Island, NY, United States), supplemented with 15% FBS (v/v; HyClone, Logan, Utah, United States), 1% nonessential amino acids (NEAA; v/v; Gibco, Grand Island, NY, United States), 2 mM GlutaMAX (Gibco, Grand Island, NY, United States), and bFGF (Life Technologies, Gaithersburg, MD, United States). PFF cells were seeded 1 day prior to transfection in 6-well plates (Corning, Corning, NY, United States). Four microgram base editor vector (hA3A-BE3 or hA3A-BE3-NG) and 2.73 μg pGL3-U6-sgRNA-tdTomato were co-transfected into 5 × 10^5^ PFF cells by nucleofection with Lonza/Amaxa Nucleofector 2B (Lonza, Basel, Switzerland) according to the manufacturer’s recommended protocol. Cells were harvested approximately 48 h post-transfection. PFF cells were cultured at 38.5°C with 5% of CO_2_.

### Fluorescence-Activated Cell Sorting

HEK293T and PFF cells were harvested and subjected to flow cytometry 48 h after transfection. A total of 10,000 cell events were collected and analyzed using FlowJo software. Single PFF cell with positive signal was seeded into 96-cell plates and cultured for 8 days to form colonies.

### Base Editing Analysis and Single Cell Line Genotyping

Genomic DNA of HEK293T and PFF cells was extracted using One Step Mouse GenoTyping Kit (Vazyme, Nanjing, China). The cell lysate was then used as the PCR template. PCR fragments for Sanger sequencing were generated in one step PCR reaction. The editing efficiency was analyzed by an online tool, EditR 1.0.9.^1^ The primers are listed in Supplementary Table S3.

### Reverse Transcription-PCR

Total RNA was extracted from cultured cells by using TRIzol reagent (Invitrogen, Carlsbad, CA, United States), according to manufacturer’s protocol. Complementary DNA (cDNA) was generated by using Thermo Scientific RevertAid First Strand cDNA Synthesis Kit (Thermo Fisher Scientific, Waltham, MA, United States). The PCR reaction with 25 ng cDNA template was performed for 30 cycles. The housekeeping gene, *GAPDH*, was used as an internal control. Relative expression of Cas9 was detected by gel electrophoresis. All the primer sequences were shown in Supplementary Table S4.

### Statistical Analysis

The statistical data are expressed as mean ± SEM, and at least three individual replicates were conducted in all experiments. Statistical significance was analyzed with unpaired Student’s *t*-tests using GraphPad prism software 6.0 (GraphPad Prism, La Jolla, CA, United States). A value of *p* < 0.05 was considered statistically significant.

## Results

### The Successful Construction of hA3A-BE3-NG Targeting Plasmid

**Figure 1:**
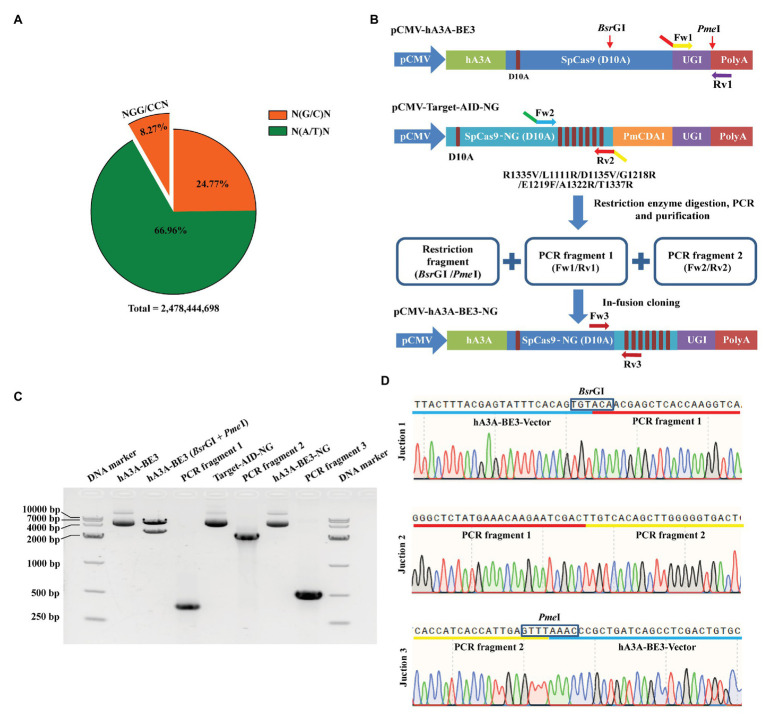
Construction of hA3A-BE3-NG vector for expanded targeting scope. **(A)** Pie chart shows the proportion of porcine genomic sites that can be targeted by SpCas9 or SpCas9-NG with distinct protospacer adjacent motif (PAM) specificities (NGG or NG). Pig reference genome (Sscrofa11.1) was used for analysis. **(B)** Schematic of the pCMV-hA3A-BE3-NG vector. Compared to SpCas9 (D10A) in hA3A-BE3, SpCas9-NG (D10A) in Target-AID-NG contained seven amino acids variants: R1335V, L1111R, D1135V, G1218R, E1219F, A1322R, and T1337R. hA3A-BE3-NG was constructed by in-fusion cloning of a restriction fragment digested *via Bsr*GI and *Pme*I from hA3A-BE3, PCR fragment 1 amplified from hA3A-BE3 *via* Fw1/Rv1 primers, and PCR fragment 2 amplified from Target-AID-NG *via* Fw2/Rv2 primers. Overlapping sequences exist in the junction of the three different fragments. **(C)** The gel image indicates that hA3A-BE3 was digested into two fragments by *Bsr*GI and *Pme*I. The PCR fragment 1 (387 bp) was amplified from hA3A-BE3 *via* Fw1/Rv1 primers, and the PCR fragment 2 (2,556 bp) was amplified from Target-AID-NG *via* Fw2/Rv2 primers. The large fragment (5,570 bp) from hA3A-BE3, PCR fragment 1 and PCR fragment 2 were fused into a recombinant vector, hA3A-BE3-NG, which was confirmed by a PCR product (540 bp) amplified *via* Fw3/Rv3 primers. **(D)** The chromatograms of Sanger sequencing show the junctional sequence was accurate among the above three fragments in recombinant hA3A-BE3-NG.

The targetable scope of traditional BEs was restricted for the conventional SpCas9 preferred to recognize the target loci with NGG PAM. Approximately 205,013,891 NGG and CCN sites exist in the pig genome, which accounts for only 8.27% of the total genome sites (Figure 1A) within the approximately 2,478,444,698 base pairs estimated by Sscrofa11.1 assembly (Li et al., 2017). Overall, the percentage of NGN and NCN sites in the porcine genome was about 33.04%, which is four times higher than that of NGG and CCN sites (Figure 1A). To expand the targeting scope of hA3A-BE3, we fused SpCas9-NG with hA3A-BE3 to generate a new BE named hA3A-BE3-NG by in-fusion strategy (Figure 1B and Supplementary Figure S1). Our construct incorporated three fragments: a restriction fragment of 5,570 bp digested by *Bsr*GI and *Pme*I from hA3A-BE3, and two PCR fragments amplified from hA3A-BE3 and Target-AID-NG, respectively (Figures 1B,C). The successful construction of the vector was confirmed by PCR, gel electrophoresis (Figure 1C), and Sanger sequencing (Figure 1D).

### hA3A-BE3-NG-Mediated Gene Editing at NGN PAM Sites in Human Cells

**Figure 2:**
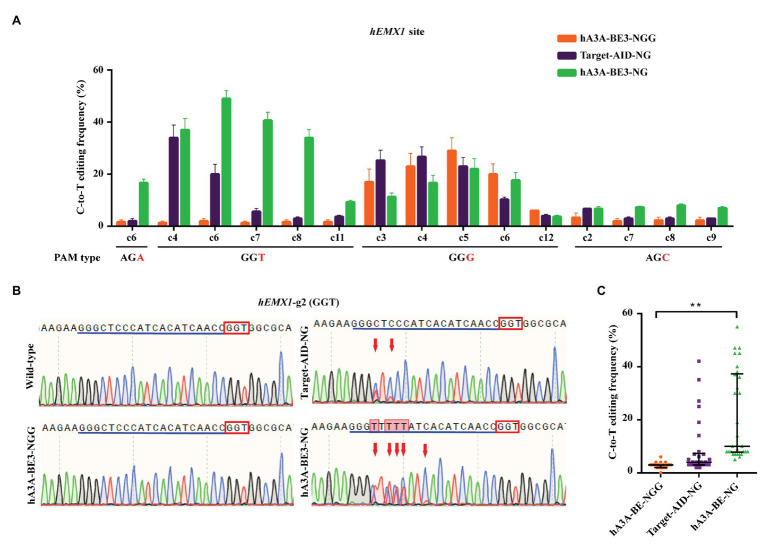
Efficient C-to-T conversion in human HEK293T cells by hA3A-BE3-NG. **(A)** C-to-T editing by hA3A-BE3, hA3A-BE3-NG, and Target-AID-NG at four endogenous *EMX1* gene sites in human HEK293T cells. The target base in the editing window is shown, counting the end distal to the PAM as position 1. Data are represented as the mean ± SEM (*n* = 3). **(B)** Sanger sequencing results of HEK293T cells transfected with hA3A-BE3, hA3A-BE3-NG, or Target-AID-NG. Red boxes indicate PAMs and blue lines indicate sgRNA sequences. Red arrows indicate substituted nucleotides. **(C)** Statistical analysis of the C-to-T editing frequency induced by hA3A-BE3, hA3A-BE3-NG, or Target-AID-NG at NGH PAM sites in **(A)**. The median and interquartile range (IQR) are shown; ^**^ *p* < 0.01.

One study revealed that Target-AID-NG was another superior base editor for introducing C-to-T conversion at NGN PAM sites efficiently in human cells (Nishimasu et al., 2018). To further validate the editing capacity of hA3A-BE3-NG, fused with different cytosine deaminase, four sgRNAs that targeted AGA, GGT, GGG, and AGC PAMs sites in *human empty spiracles homeobox 1* (*EMX1*) loci were designed. The hA3A-BE3, hA3A-BE3-NG, or Target-AID-NG plasmid were co-transfected with sgRNAs‐ and GFP-expressing plasmids into HEK293T cells, respectively. All GFP-positive cells (no less than 25% of total cells) were isolated *via* flow cytometry for further characterization (Supplementary Figures S2A,B). The expression of hA3A-BE3-NG was confirmed by reverse transcription PCR (RT-PCR) in 48 h post-transfected HEK293T cells (Supplementary Figure S2C). Mutation frequencies by different BEs at NGN PAM sites were quantified using Sanger sequencing and EditR software (Figures 2A–C and Supplementary Figure S2D). Results showed that hA3A-BE3-NG achieved a C-to-T editing frequency of at least 15% at AGA and GGT PAM sites when compared with the mutation frequency of hA3A-BE3 showed less than 5% (Figures 2A,B and Supplementary Figure S2D). The hA3A-BE3-NG induced slightly lower C-to-T conversion, compared to those of hA3A-BE3 (14.27 vs. 19.00%) in the activity window (C3, C4, C5, C6, and C12) at GGG PAM (Figure 2A and Supplementary Figure S2D). This is in line with a recently observed phenomenon, SpCas9-NG shows slightly reduced activity at NGG PAM sites in human cells (Nishimasu et al., 2018). In addition, hA3A-BE3-NG also showed relatively low conversion efficiency at AGC PAM sites (Figure 2A and Supplementary Figure S2D), which is consistent with a previous report (Nishimasu et al., 2018). Overall, hA3A-BE3-NG-mediated C-to-T conversion was more efficient than hA3A-BE3 at NGH PAM sites (21.27 vs. 2.81% at average; Figure 2C). In addition, Target-AID-NG showed efficient editing of C3 and C4 at GGG PAM site (Figure 2A and Supplementary Figure S2D), highlighting differences in editing windows, base preference, and efficiencies between hA3A‐ and PmCDA1-derived BEs. However, hA3A-BE3-NG achieved a higher mutation frequency than that of Target-AID-NG at AGA and GGT PAM sites (Figures 2A,B and Supplementary Figure S2D), indicating that hA3A-BE3-NG could be considered a more efficient BE with an expanded targetable scope for gene editing in the mammalian genome.

### Expanded Editable Scope Through hA3A-BE3-NG to Generate Premature Terminal Codon in PFF Cells

**Figure 3:**
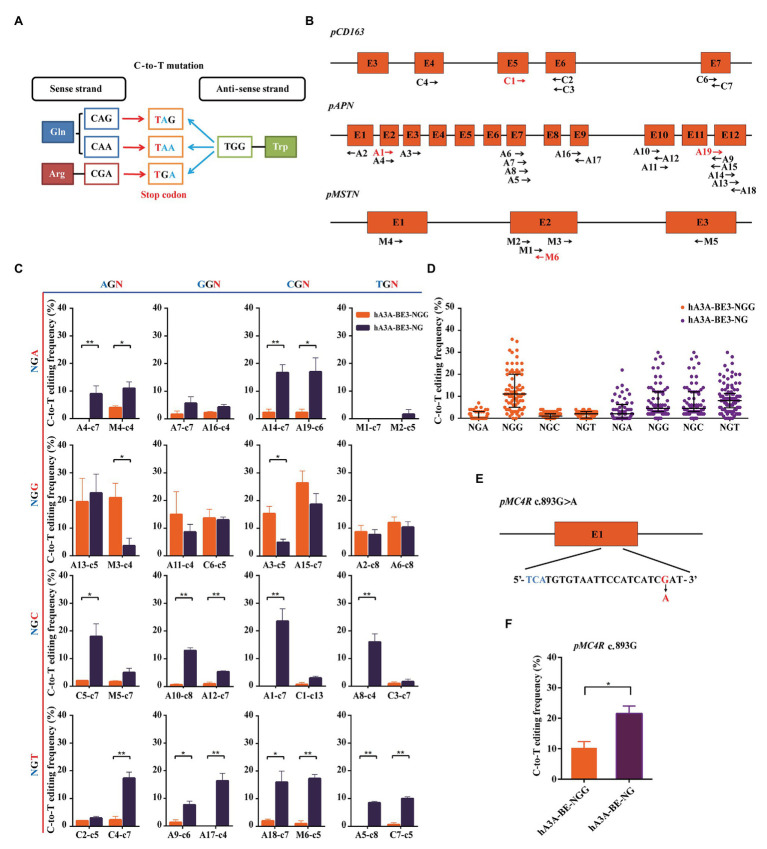
Precision missense mutation using hA3A-BE3-NG to expand the editing scope in porcine fetal fibroblast (PFF) cells. **(A)** Representation of the C-to-T conversion induced by base editors to generate stop codons. The base editors convert CAA, CAG, and CGA codons to stop codons (red) in the sense strand. The TGG codon is converted to stop codons (blue) through G-to-A conversion. **(B)** Schematic of the target sites at the porcine *CD163*, *APN*, and *MSTN* loci. The target sites indicated by the black arrows can generate stop codons using base editors (BEs). The forward direction of arrow indicates sgRNA-matched anti-sense strand, and vice versa. Total of 32 sgRNAs were designed (A1–19, C1–7, and M1–6). **(C)** Base editing at 32 NGN PAM sites by hA3A-BE3 and hA3A-BE3-NG. The target sites covered all 16 possible NGN PAM combinations, counting the end distal to the PAM as position 1. **(D)** Statistical analysis of the C-to-T editing frequency induced by hA3A-BE3 or hA3A-BE3-NG at a total of 32 endogenous target sites. The median and IQR are shown. **(E)** Schematic of the target site at *MC4R* locus. *MC4R* c.893G>A could be produced by hA3A-BE3-NG. The PAM sequence and substituted base are shown in blue and red, respectively. **(F)** Base editing at the *MC4R* locus by hA3A-BE3 and hA3A-BE3-NG. In **(C,F)** values were shown as mean ± SEM (*n* = 3); ^**^ *p* < 0.01 and ^*^ *p* < 0.05.

Stop codons (TAG, TGA, or TAA) could be produced by a C-to-T conversion of the CAG, CGA, or CAA codons on the sense strand and the G-to-A conversion of the TGG codon caused by C-to-T mutation on the anti-sense strand (Figure 3A). The loss of function mutation in various genes was reported to confer the elite traits in pigs, such as *CD163* gene for PRRSVs resistant (Whitworth et al., 2016; Burkard et al., 2017; Wells et al., 2017), *APN* gene for TGEVs resistant (Luo et al., 2019; Whitworth et al., 2019; Zhang et al., 2019), and *MSTN* for increased lean meat production (Qian et al., 2015; Wang K, et al., 2015). Thus, we explore the possibility and editing efficiency of inducing stop codons over these loci at expanded targetable sites by hA3A-BE3-NG in pigs. We designed a total of 32 sgRNAs (A1–19, C1–7, and M1–6) with NGN PAM in porcine *CD163*, *APN*, and *MSTN* genomic loci. Of the 32 sgRNAs, 28 (A2–18, C2–7, and M1–5) could produce premature terminal codons in the targeted activity windows if C-to-T conversion occurs (positions 2–13, counting the PAM as positions 21–23; Figure 3B). We firstly evaluated hA3A-BE3-NG-mediated editing efficiency on 32 NGN PAM sites (Supplementary Figure S3A). hA3A-BE3-NG showed comparable activity to hA3A-BE3 at 6 NGG PAM sites (A2, A6, A11, A13, A15, and C6) and reduced activity at 2 NGG PAM sites (A3 and M3), suggesting that hA3A-BE3-NG was also a useful BE at NGG PAM sites in pigs (Figure 3C and Supplementary Figure S3B). With NGH PAM sites, hA3A-BE3-NG showed at least a 3% mutation frequency at 21 of the 24 sites and at least a 10% mutation frequency at half of the 21 sites (Figure 3C and Supplementary Figure S3C). By contrast, hA3A-BE3 only edited the AGA PAM site (M4) with a low mutation frequency of 4% and had no efficiency at other 23 NGH PAM sites (Figure 3C). Interestingly, as shown in Figure 3C, hA3A-BE3-NG was editing ineffective at modifying TGA PAM sites (M1 and M2), which might be resulted from sequence signatures and nucleotide preferences (Xue et al., 2019). In brief, compared with hA3A-BE3 that induced C-to-T conversion efficiently at NGG PAM sites, hA3A-BE3-NG showed efficient editing at a variety of PAM sites (Figures 3C,D and Supplementary Figures S3B,C). Particularly, at 25 of 28 target sites that sgRNAs could generate premature stop codons to knockout target genes, hA3A-BE3-NG achieved detectable C-to-T mutation frequency if 3% was used as the cutoff threshold. By contrast, only 9 of these 28 sgRNAs were functional with hA3A-BE3 (Figure 3C). Besides the above three genes that was designed to induce loss-of-function mutations, we further exploited to introduce a beneficial SNP (c.893 G>A) into *MC4R* gene that was reported to be a marker for decreased fat deposition trait (Kim et al., 2000; Schroyen et al., 2015). The sgRNA was designed on the reverse strand of TGA PAM site, positioning the targeted cytosine in the activity window of hA3A-BE3-NG to produce *MC4R* c.893G>A on the sense strand (Figure 3E). hA3A-BE3-NG mediated higher mutation frequency than hA3A-BE3 (21.67 vs. 10.33%; Figure 3F and Supplementary Figure S3D).

### hA3A-BE3-NG-Mediated Base Editing in Multiple Loci

**Figure 4:**
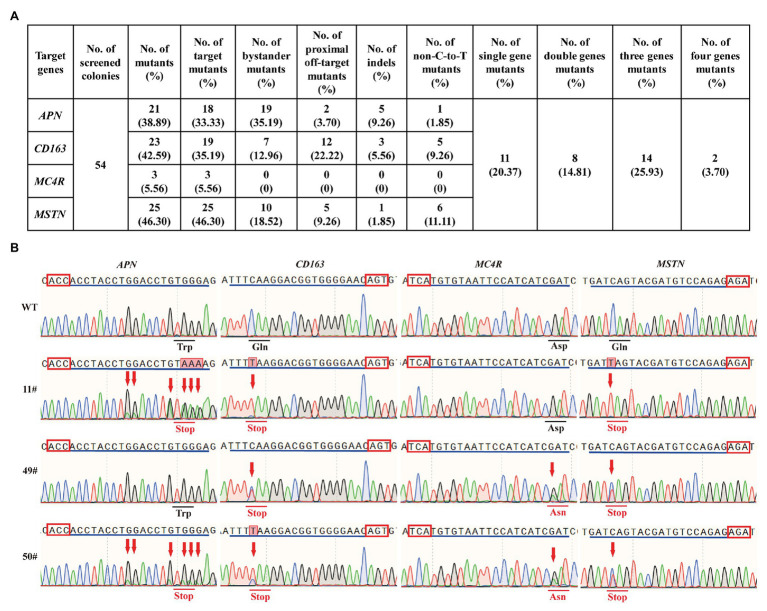
hA3A-BE3-NG-mediated base editing at multiple genes in PFF cells. **(A)** Summary of multiple sites base editing by hA3A-BE3-NG in PFF cells. **(B)** Sanger sequencing results of selected single-cell colonies. 11# and 49# colonies have mutations on three genes, and 50# colony has mutations on all four genes. The red box indicates the PAMs and the blue line indicates the sgRNA sequence. The red arrow indicates the substituted nucleotide. The amino acid in the red line indicates expected substitutions at target sites.

In livestock, most of the economic traits were considered to be regulated by a massive number of SNPs in various genes (Song et al., 2020). Thus, the ability to create precise and multiple genetic modification in various loci across the pig genome simultaneously is necessary for successful pyramid breeding. To investigate the feasibility of hA3A-BE3-NG for base editing in multiple loci, we simultaneously co-transfected hA3A-BE3-NG and sgRNAs-tdTomato-expressing plasmid that targeted *APN*, *CD163*, *MC4R*, and *MSTN* into PFF cells. After 48 h of transfection, tdTomato-positive single PFF cell was isolated and seeded into 96-cell plates *via* FACS, and then cultured for another 8 days to form single-cell colonies. A total of 54 colonies were obtained and genotyped by Sanger sequencing (Figures 4A,B). Results showed that 21 out of 54 (38.89%), 23 out of 54 (42.59%), 3 out of 54 (5.56%), and 25 out of 54 (46.30%) colonies had mutations in the *APN*, *CD163*, *MC4R*, and *MSTN* genes, respectively, and most of them had effective C-to-T conversion at the target sites (Figure 4A). Due to the wide activity window of hA3A-BE3-NG, we also found that a number of colonies had bystander mutations with C-to-T substitution existing in the vicinity of the targeted cytosine (35.19, 12.96, and 18.52% colonies in *APN*, *CD163*, and *MSTN* genes, respectively; Figure 4A). Moreover, 12 out of 54 (22.22%) colonies showed a proximal off-target mutation at position −4 (with the base distal from the PAM set as position 1) in *CD163* (Figure 4A). Importantly, 35 out of 54 colonies had mutations, and therein, two single-cell colonies (3.70%, 2/54) showed targeted mutations of all four genes (*APN*, *CD163*, *MSTN*, and *MC4R*; Figures 4A,B). In addition, we identified 14 colonies (25.93%, 14/54) with triple-gene mutations, 8 (14.81%, 8/54) with double-gene mutations, and 11 (20.37%, 11/54) with single-gene mutation (Figures 4A,B).

## Discussion

Genome editing technologies have provided a revolutionary strategy for making genetic improvements in pig breeding. Compared to conventional cross breeding in livestock, the molecular breeding to accurately modify the agriculture-related functional genes will save a lot of time, money, and manpower. The focus of recent genome editing research is to modify the genome efficiently, accurately, and safely. In the current study, hA3A-BE3-NG was constructed and proved to be a powerful base editor to improve the editing efficiency and expand the targeting scope in pigs. It has been reported that, 20 endogenous target sites (including *EMX1*, *VEGFA*, *GRIN2B*, etc.) with different PAM have been used to compare C-to-T conversion efficiency between Target-AID and Target-AID-NG in HEK293T cells (Nishimasu et al., 2018). Therefore, we also selected the *EMX1* targets from the study above to analyze the targeting efficiency of hA3A-BE3, Target-AID-NG, and hA3A-BE3-NG in the current study. Here, hA3A-BE3-NG was confirmed to show editing activity comparable with or even higher than Target-AID-NG at the four target sites in human cells. And it could induce C-to-T mutation in a broader activity window in human and porcine cells efficiently, which is consistent with a previous study that hA3A-BE3 had an approximately 12 nucleotides activity window (Wang et al., 2018).

To avoid potential chimeric issues and long-time frame of breeding, the generation of genetically modified large animals was mostly created by genome editing technology combined with somatic cell nuclear transfer (SCNT) instead of embryo injection (Zhao et al., 2019). So how to obtain the cell colonies with desired modification efficiently is one of the key steps. Here, we found that hA3A-BE3-NG could induce C-to-T conversion efficiently not only at NGG PAM sites as hA3A-BE3 but also exhibited expand targeting scope at NGN PAM sites. For the *MC4R* c.893G>A mutation, hA3A-BE3-NG showed more efficient than hA3A-BE3 at the TGA PAM site (21.67 vs. 10.33% at average). Thus, applications of hA3A-BE3-NG could expand the editing scope at NGN PAM sites, possibly facilitating breeding improvements in pigs.

With the development of functional genomics and bioinformatics, more and more SNPs responsible for economic traits have been identified in livestock (Song et al., 2020). And many economic traits are majorly controlled or orchestrated by combinations of SNPs. Therefore, it is of importance to create precise and multiple genome-editing livestock for exploring the function of SNPs and evaluate their potential breeding value. In addition, the potential of chromosomal structural abnormalities would increase when multiplex target loci were cut simultaneously by conventional CRISPR-Cas systems, causing genomic instability, chromosome elimination, and even cell death (Wang T, et al., 2015; Aguirre et al., 2016; Zuo et al., 2017). BEs provided a safe strategy to edit multiple gene sites efficiently and accurately without DSBs. Recently, the multiplex base editing was accomplished by BE3 at NGG PAM sites in pigs (Xie et al., 2019; Yuan et al., 2019). Using BE3 and hA3A-BE3, Xie et al. (2019) simultaneously mutated the porcine *RAG1*, *RAG2*, and *IL2RG* or *DMD*, *TYR*, and *LMNA* triple gene in PFF cells with high efficiency, and subsequently generated a triple gene knockout pig model with immunodeficiency for applications in regenerative medicine. Yuan et al. (2019) prepared *GGTA1/B4GAlNT2/CMAH* triple gene knockout pigs which could be used as organ donors for xenotransplantation by BE4-Gam. hA3A-BE3-NG could simultaneously introduce targeted mutations at multiple sites of four genes, *APN*, *CD163*, *MSTN*, and *MC4R* in PFF cells, suggesting the great potential of hA3A-BE3-NG in animal pyramid breeding.

Previous studies have suggested that CBEs could cause DNA off-target effects in mouse embryos and plants (Jin et al., 2019; Zuo et al., 2019); however, BE variants are continuously being improved and exploited to improve targeted specificity (Doman et al., 2020). In this current study, bystander and proximal off-target mutations were also found at *APN*, *CD163*, and *MSTN* gene sites, resulting from the wide editing window of hA3A-BE3-NG. Some engineered precise hA3A variants have been developed to reduce bystander mutations such as hA3A-Y130F *via* narrowing the width of the editing window and eA3A (hA3A-N57G) according to the preferential target base motif (Gehrke et al., 2018; Wang et al., 2018; Liu et al., 2020a). These off-target effects are less crucial when using base editing to introduce premature terminal codons, generating loss-of-function mutations and inactivating protein function. In agricultural breeding, the unpredicted editing byproducts through BEs might be more tolerated and could provide a new source of mutations with favorable economic characteristics. Recently, it has been reported that some new engineering variant of the Cas9, SpRY, which is free of PAM restriction (Walton et al., 2020). In the future, the combine of BEs and the new Cas9 variant will further expand the editing scope to improve base editing tools for pyramid breeding and genetic improvement in livestock.

In summary, we generated hA3A-BE3-NG, a versatile CBEs, that substantially expands the scope and capability of base editing at NGN PAM sites. To our knowledge, this is the first study to precisely edit multiple genes responsible for economic traits in the porcine genome using BEs, suggesting the incredible potential of using BEs to accelerate molecular pyramid breeding in livestock.

## Data Availability Statement

The original contributions presented in the study are included in the article/Supplementary Material and further inquiries can be directed to the corresponding authors.

## Ethics Statement

The animal study was reviewed and approved by Laboratory Animal Welfare and Ethics Committee Institute of Zoology, Chinese Academy of Sciences.

## Author Contributions

All authors listed have made a substantial, direct and intellectual contribution to the work, and approved it for publication.

### Conflict of Interest

The authors declare that the research was conducted in the absence of any commercial or financial relationships that could be construed as a potential conflict of interest.

## Footnotes

^1^https://moriaritylab.shinyapps.io/editr_v10/
