# Supplementary material for: Cytosine Base Editor (hA3A-BE3-NG)-Mediated Multiple Gene Editing for Pyramid Breeding in Pigs
Source: Front Genet. 2020 Nov 16;11:592623. doi: 10.3389/fgene.2020.592623 (PMC7701296; doi:10.3389/fgene.2020.592623)

**Supplementary Material**

Additional supporting information may be found online in the Supplementary Material section at the end of the article.

**Supplementary Table S1** sgRNAs used in this study.

**Supplementary Table S2** Primers used for constructing the hA3A-BE3-NG plasmid.

**Supplementary Table S3** Primers used for genotyping.

**Supplementary Table S4** Primers used for RT-PCR.

**Supplementary Figure S1** Sequence analysis of between hA3A-BE3 and Target-AID-NG.

**Supplementary Figure S2** Base editing analysis of transfected HEK293T cells.

**Supplementary Figure S3** Expanded editable scope through hA3A-BE3-NG in PFF cells.

**Supplementary Table S1.** sgRNAs used in this study

| Site | Protospacer sequence (5’-3’) | PAM |
| --- | --- | --- |
| sgRNA-hEMX1-g1 | AGGACAAAGTACAAACGGC | AGA |
| sgRNA-hEMX1-g2 | GGCTCCCATCACATCAACC | GGT |
| sgRNA-hEMX1-g3 | GCCCCAGTGGCTGCTCTGG | GGG |
| sgRNA-hEMX1-g4 | CTTTACCCAGTTCTCTGGG | AGC |
| sgRNA-pAPN-g1 | GCAAGCCGTGGAACCGGTAC | CGC |
| sgRNA-pAPN-g2 | ACCGGTTCCACGGCTTGCTC | TGG |
| sgRNA-pAPN-g3 | GTTCCAGAGCGTGAATGAAA | CGG |
| sgRNA-pAPN-g4 | ACGACACAGATGCAGTCTAC | AGA |
| sgRNA-pAPN-g5 | ACCTGCCCAGATCAGCGAGA | TGT |
| sgRNA-pAPN-g6 | CGTGTACCGAGTGATGGCTG | TGG |
| sgRNA-pAPN-g7 | GTGTACCGAGTGATGGCTGT | GGA |
| sgRNA-pAPN-g8 | TACCGAGTGATGGCTGTGGA | TGC |
| sgRNA-pAPN-g9 | AAGACCCAATCGTCCGATGC | GGT |
| sgRNA-pAPN-g10 | GGATGCTCAGACGTCCATCA | GGT |
| sgRNA-pAPN-g11 | GATCGATGGACCCTGCAGAT | GGG |
| sgRNA-pAPN-g12 | AGGGTCCATCGATCCATGAT | GGC |
| sgRNA-pAPN-g13 | TTTCCAGGTGAACTACGACG | AGG |
| sgRNA-pAPN-g14 | TATTTCCAGGTGAACTACGA | CGA |
| sgRNA-pAPN-g15 | CAAGACCCAATCGTCCGATG | CGG |
| sgRNA-pAPN-g16 | TATCAGAACACCACCTACCT | GGA |
| sgRNA-pAPN-g17 | TCCCACAGGTCCAGGTAGGT | GGT |
| sgRNA-pAPN-g18 | CATCCTCCAGTTGTCCTCGT | CGT |
| sgRNA-pAPN-g19 | TTGTTCAAAACCGCATCGGA | CGA |
| sgRNA-pMSTN-g1 | GGAATCCGATCTCTGAAACT | TGA |
| sgRNA-pMSTN-g2 | GGCCCAACTGTGGATATATC | TGA |
| sgRNA-pMSTN-g3 | AAACAACCTGAATCCAACTT | AGG |
| sgRNA-pMSTN-g4 | GATCAGTACGATGTCCAGAG | AGA |
| sgRNA-pMSTN-g5 | ATAATCCAGTCCCATCCAAA | AGC |
| sgRNA-pMSTN-g6 | GATTCCAGTATACCTTGTAC | CGT |
| sgRNA-pCD163-g1 | ACTGCCATTGGTCGAGTTAA | CGC |
| sgRNA-pCD163-g2 | ACCCCACTGCCAATTCTTGC | AGT |
| sgRNA-pCD163-g3 | TCCACCCCACTGCCAATTCT | TGC |
| sgRNA-pCD163-g4 | TTTCAAGGACGGTGGGGAAC | AGT |
| sgRNA-pCD163-g5 | TCTTGTCGAGGGAATGAGTC | AGC |
| sgRNA-pCD163-g6 | AGTACAACATGGAGACACGT | GGG |
| sgRNA-pCD163-g7 | TGCCCCACGTGTCTCCATGT | TGT |
| sgRNA-pMC4R-g1 | ATCGATGATGGAATTACACA | TGA |

**Supplementary Table S2.** Primers used for constructing the hA3A-BE3-NG plasmid

| Primer name | Primer sequence (5’-3’) | Product size (bp) |
| --- | --- | --- |
| hA3A-NG-Fw1 | TATTTCACAGTGTACAACGAGCTCACCAAGGTCAA | 2556 |
| hA3A-NG-Rv1 | GTCACCCCCAAGCTGTGACAAGTCGATTCTTGTTTCATAGA |  |
| hA3A-NG-Fw2 | TCTATGAAACAAGAATCGACTTGTCACAGCTTGGGGGTGAC | 387 |
| hA3A-NG-Rv2 | GAGGCTGATCAGCGGGTTT |  |
| hA3A-NG-Fw3 | GACAACGGTAGCATTCCACA | 540 |
| hA3A-NG-Rv3 | ATCCTCCACTCCGCTGATTT |  |

**Supplementary Table S3.** Primers used for genotyping

| Primer name | Primer sequence (5’-3’) | Product size (bp) |
| --- | --- | --- |
| pAPN-e1-GT-F1 | TGGCCAAGGGATTCTACATT | 451 |
| pAPN-e1-GT-R1 | ACCAGCTCAGTCCTGTCGAT |  |
| pAPN-e2a3-GT-F1 | CTGTGCACCCCTTTCCAT | 691 |
| pAPN-e2a3-GT-R1 | TGCAGAGACTGAAGGTCAGAGA |  |
| pAPN-e7-GT-F1 | GAATCTGGTAAGCCCACTGC | 461 |
| pAPN-e7-GT-R1 | CAGGAAGTTGGAGAGCATCC |  |
| pAPN-e9-GT-F1 | GAGCCTCGGTTATCAGGATG | 275 |
| pAPN-e9-GT-R2 | TGGTGGCTGGCTATCACTGA |  |
| pAPN-e10-GT-F1 | GCACTCTTGGCTCATCCAGT | 468 |
| pAPN-e10-GT-R1 | CAGACCCTGCTATGGTCAGA |  |
| pAPN-e12-GT-F1 | AAGGTAAGCCCCTCTCCTTG | 436 |
| pAPN-e12-GT-R1 | TAGATGACCTGAGCCCGATT |  |
| pCD163-e3-GT-F2 | GGGTGGTGAAAACAAGTGCT | 469 |
| pCD163-e3-GT-R2 | TGGGCTTTGTCATGTGTAAA |  |
| pCD163-e4-GT-F2 | TGATTTAGAGATGAGGCTGGTG | 585 |
| pCD163-e4-GT-R2 | GGGAATGGGCTATTCTCTGA |  |
| pCD163-e5-GT-F2 | TCCCCTTTTCACTCACTCCTC | 443 |
| pCD163-e5-GT-R2 | GGTCCTTCCGGTGTTTTGTTTTC |  |
| pCD163-e6-GT-F2 | TTCAAAAACCCAGGAGATGG | 537 |
| pCD163-e6-GT-R2 | AAGTGGTGCTGGGAAAACTG |  |
| pCD163-e7-GT-F1 | GGCAAAGTCCCAGAATTGTC | 597 |
| pCD163-e7-GT-R1 | TGGAAGGAGACAGGGCTTTA |  |
| pMSTN-e1-GT-F2 | GCCTGGAAACAGCTCCTAAC | 483 |
| pMSTN-e1-GT-R2 | CCTTGGTGGCATTAAGCTGT |  |
| pMSTN-e2-GT-F1 | TGGATGTTCCTCCACAGTGTC | 858 |
| pMSTN-e2-GT-R1 | CAGGGCTACCATTGGGGTAA |  |
| pMSTN-e3-GT-F1 | GGGTAGGAAAGTGATTCAGGA | 354 |
| pMSTN-e3-GT-R1 | TGGAGACATCTTTGTGGGAGT |  |
| hEMX1-GT1-F1 | CTCACTCCATCCATCGAGGC | 465 |
| hEMX1-GT1-R1 | CAGAGTTGCGGGTGAGGAAT |  |
| hEMX1-GT2-F1 | CACTTTAGGCCCTGTGGGAG | 812 |
| hEMX1-GT2-R1 | CCCATTGCTTGTCCCTCTGT |  |

**Supplementary Table S4.** Primers used for RT-PCR

| Primer name | Primer sequence (5’-3’) | Product size (bp) |
| --- | --- | --- |
| Cas9-NG-RT-F2 | CGAGATCATCGAGCAGATCA | 158 |
| Cas9-NG-RT-R2 | CTCCCAGATTGGTCAGGGTA |  |
| hGAPDH-RT-F1 | AATCAAGTGGGGCGATGCTG | 158 |
| hGAPDH-RT-R1 | GGTTCACACCCATGACGAAC |  |

**Supplementary Figure S1:** **Sequence analysis of between hA3A-BE3 and Target-AID-NG.** **(A)** The plasmid map of hA3A-BE3 (#113410) and Target-AID-NG (#119861) from Addgene. **(B)** The comparison of DNA and protein sequence between SpCas9 (D10A) in hA3A-BE3 and SpCas9-NG (D10A) in Target-AID-NG. **(C)** Alignment results showing a slight diversity of 7 amino acids, but obvious differences in DNA sequence between SpCas9 (D10A) and SpCas9-NG (D10A). Blue shade indicates the same nucleotides and red box indicates the different amino acids.


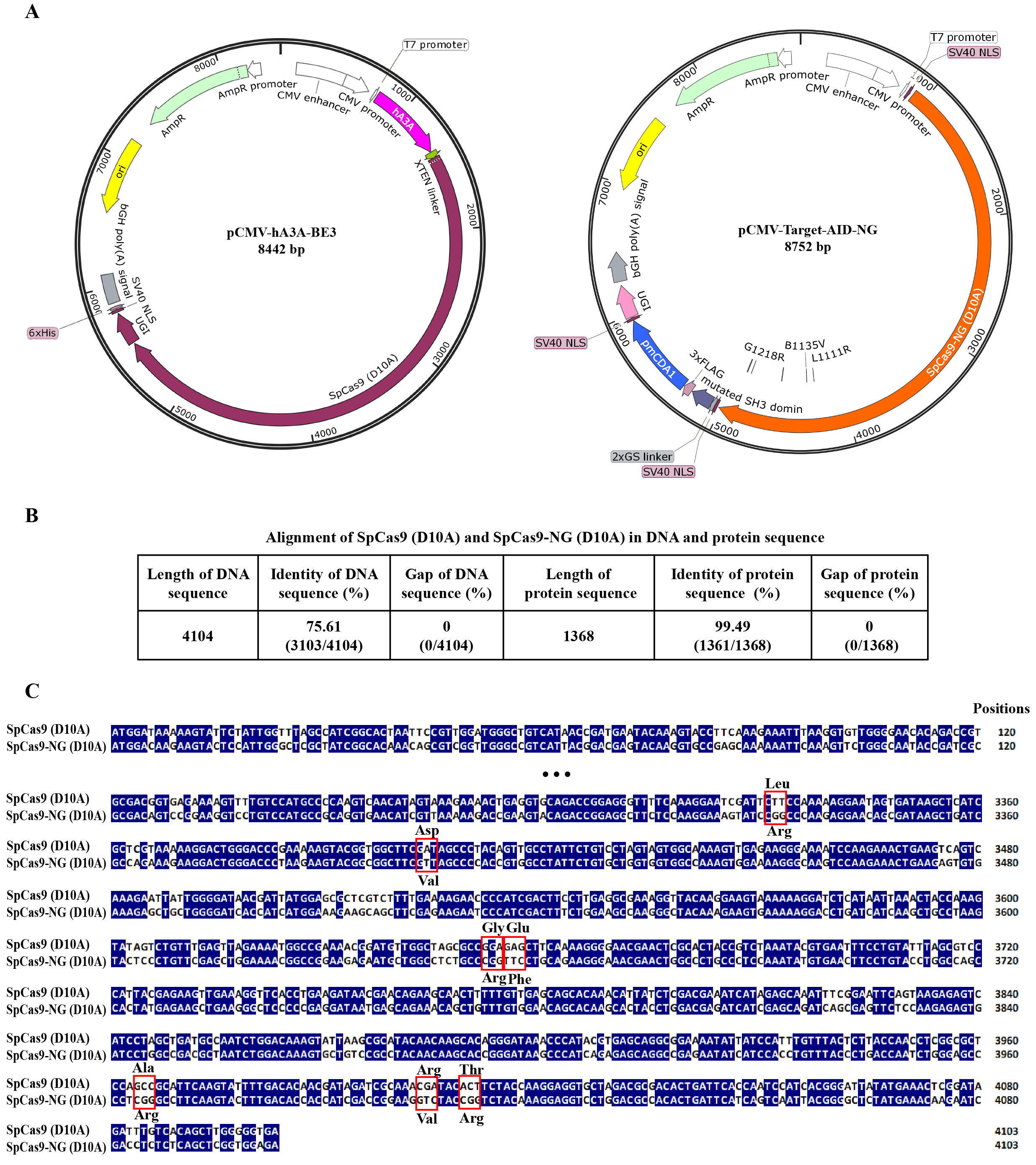


**Supplementary Figure S2:** **Base editing analysis of transfected HEK293T cells.** **(A)** The HEK293T cells were transfected with base editors, sgRNA- and GFP-expressing plasmid simultaneously. The transfected cells expressed green signal under a fluorescence microscope. Scale bars, 40 μm. **(B)** The flow cytometry showed more than 25% of transfected cells expressed GFP, and the GFP-positive cells were collected for further characterization of base-editing frequency. **(C)** RT-PCR of hA3A-BE3-NG and Target-AID co-transfected with sgRNA-hEMX1~4 (E1~E4). GAPDH was used as a control. **(D)** Sanger sequencing results of HEK293T cells transfected with hA3A-BE3, hA3A-BE3-NG or Target-AID-NG. Red boxes indicate PAMs and blue lines indicate sgRNA sequences. Red arrows indicate substituted nucleotides.


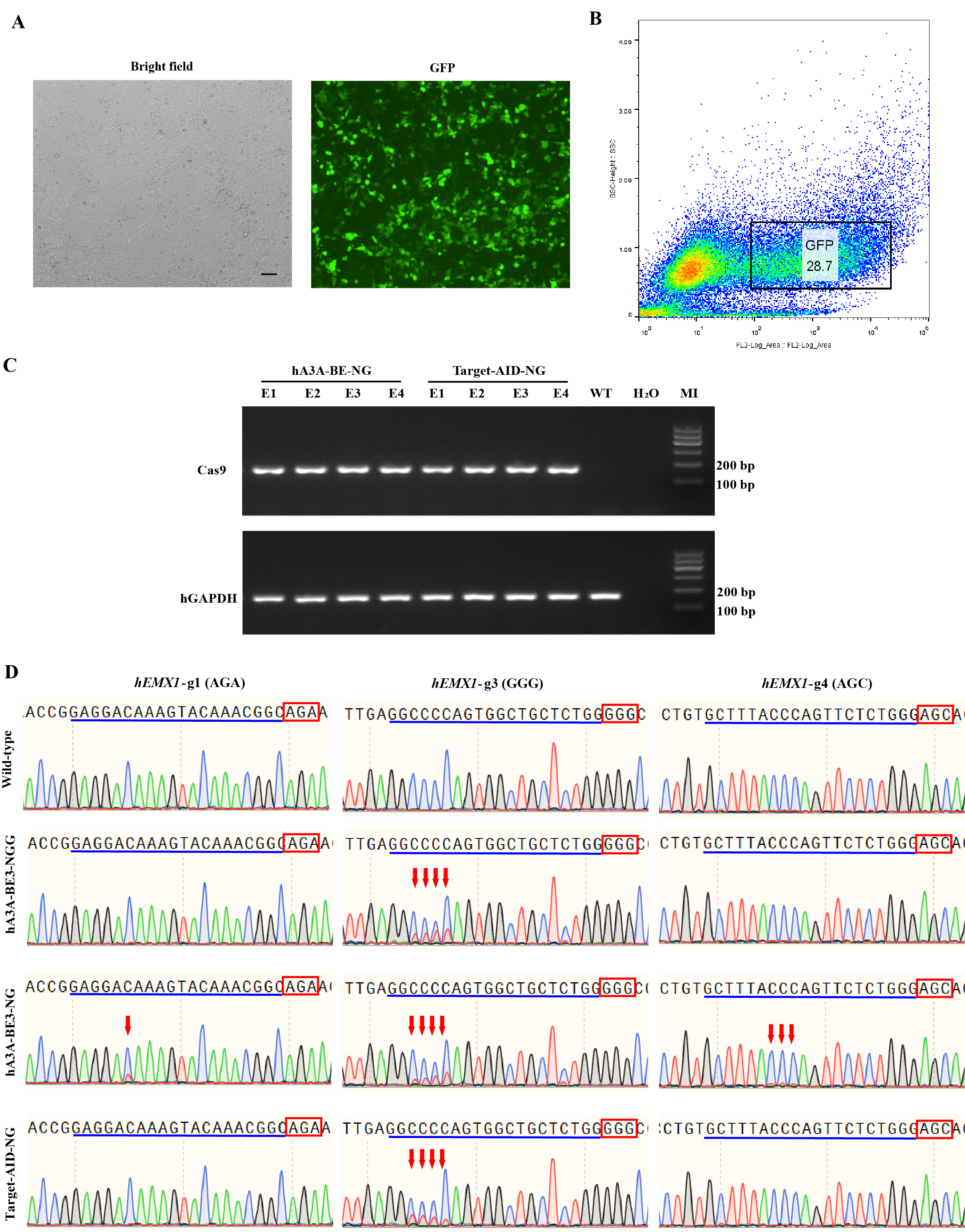


**Supplementary Figure S3:** **Expanded editable scope through hA3A-BE3-NG in PFF cells**. **(A)** The efficiency of edited Cs (positions 2-13) at targeted NGN PAM sites of hA3A-BE3-NG in PFF cells. **(B-D)** Sanger sequencing results of PFF cells transfected with hA3A-BE3 and hA3A-BE3-NG. Verification of canonical NGG PAM sites in **(B)**, non-canonical NG PAM sites in **(C)**, *MC4R* sites in **(D)** are shown by Sanger sequencing chromatograms. The red box indicates the PAMs and the blue line indicates the sgRNA sequence. The red arrow indicates the substituted nucleotide.


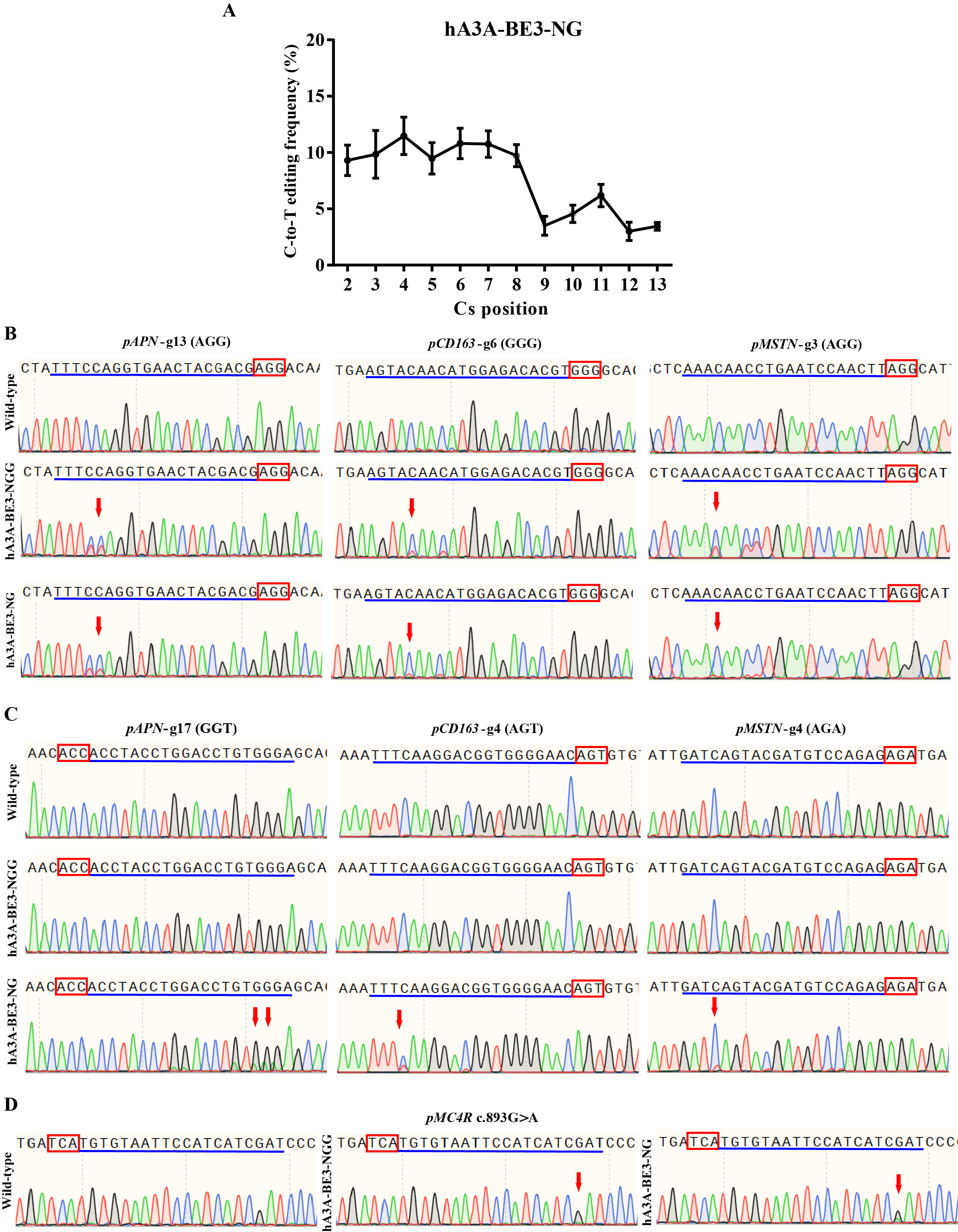

Supplement: Supplementary file 1 [file Data_Sheet_1.docx]
